# 38‐Marker Full‐Spectrum Flow Cytometry Panel for the Comprehensive Profiling of γδ T Cells in Human Blood and Lymphoid Tissues

**DOI:** 10.1002/eji.70155

**Published:** 2026-03-01

**Authors:** Mohamed Hamed, Daniela Moreno‐Vicencio, Daniel Arsovski, Mustafa Farhat, Rosie Sanders, Ellen Mann, Annabelle Bennett, Priyanka Chevour, Martin S. Davey

**Affiliations:** ^1^ Division of Biomedical Sciences Warwick Medical School University of Warwick Coventry UK; ^2^ Infection and Immunity Program and Department of Biochemistry and Molecular Biology Biomedicine Discovery Institute Monash University Clayton Australia

## Abstract

This is an update to the Guidelines for the Use of Flow Cytometry and Cell Sorting in Immunological Studies (Third Edition), Chapter 12C, by Cossarizza et al. A 38‐marker full‐spectrum flow cytometry panel enabling high‐resolution profiling of human γδ T cells across blood and lymphoid tissues, including robust identification of rare, tissue‐adapted subsets.

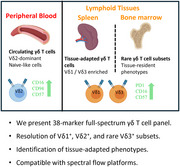

High‐dimensional profiling of tissue‐resident γδ T cells has been hampered by the lack of validated flow cytometry panels capable of resolving their diverse subsets across human tissues. Although comprehensive flow cytometry guidelines are available for immunological studies [1], the application of high‐dimensional spectral cytometry for the systematic characterisation of γδ T cells across blood and lymphoid tissues remains limited. To address this, we developed a 38‐marker spectral flow cytometry panel that enables comprehensive characterisation of γδ T cells and other key lymphocyte populations in peripheral blood and lymphoid tissues. The panel reliably identifies major γδ T cell subsets across blood, spleen, and bone marrow, while concomitantly resolving conventional αβ T cells, MAIT cells, NK cells and B cells, enabling integrated immunophenotyping across blood and lymphoid tissues.

γδ T cells are a distinct subset of T lymphocytes that exhibit both innate and adaptive immune functions. Although they represent only 1%–5% of circulating T cells [2], they are enriched at mucosal sites, where they play key roles in tissue homeostasis, immune defence and tumour surveillance. Human γδ T cells are classified based on their T cell receptor (TCR) usage into Vδ2^+^ cells, which are predominantly found in peripheral blood, and Vδ1^+^ and the rarer Vδ3^+^ subsets, which are enriched in tissues. Recent single‐cell RNA and TCR sequencing studies have revealed pronounced phenotypic and functional heterogeneity among γδ T cells, with distinct subsets defined by characteristic transcriptional and surface marker profiles [3].

Surface molecules such as CD27, CD28 and CX_3_CR1 are widely used to distinguish naïve‐like from effector‐like cells within γδ T cell populations [4]. To capture this diversity, our 38‐marker spectral flow cytometry panel incorporates markers associated with memory and effector differentiation (CD27, CD28, CD45RA, CD45RO, CD127, CX_3_CR1), activation (CD25, CD69, CD38, HLA‐DR), homing and trafficking (CCR5, CCR6, CCR7, CXCR3, CXCR5), tissue residency (CD69 and CD103) [4, 5, 6, 7] and immune checkpoint regulation (PD‐1) [8].

Using this panel, all major human lymphocyte populations were readily resolved in peripheral blood mononuclear cells (PBMCs), including γδ T cells, αβ T cells, B cells and NK cells (Figure 1A–D). γδ T cell subsets: Vδ1^+^ and Vδ3^+^ T cells were classified using CD27 and CX_3_CR1 [4] into naïve‐like (CD27^+^CX_3_CR1^−^) and effector‐like (CD27^lo^ CX_3_CR1^+^) cells. Vδ2^+^ γδ T cell differentiation stages were defined using CD27 and CD28 [9] (Figure 1B). For αβ T cells, CD4^+^ and CD8^+^ subsets were classified into naïve (CD45RA^+^ CD27^+^), central memory (CD45RA^−^ CD27^+^), effector memory (CD45RA^−^ CD27^−^) and terminally differentiated effector memory (TEMRA; CD45RA^+^ CD27^−^) subsets (Figure 1C). NK cells were subdivided into CD56^hi^ CD16^dim^, CD56^dim^ CD16^hi^ and CD56^lo^ CD16^lo^ populations, representing distinct maturation and functional NK cell states, ranging from cytokine‐producing to cytotoxic phenotypes. B cells were categorised by IgD and CD27 expression into naïve (IgD^+^ CD27^−^), unswitched memory (IgD^+^ CD27^+^), switched memory (IgD^−^ CD27^+^) and atypical (IgD^−^CD27^−^) B cell populations, reflecting stages of antigen experience and differentiation (Figure 1D).

**FIGURE 1:**
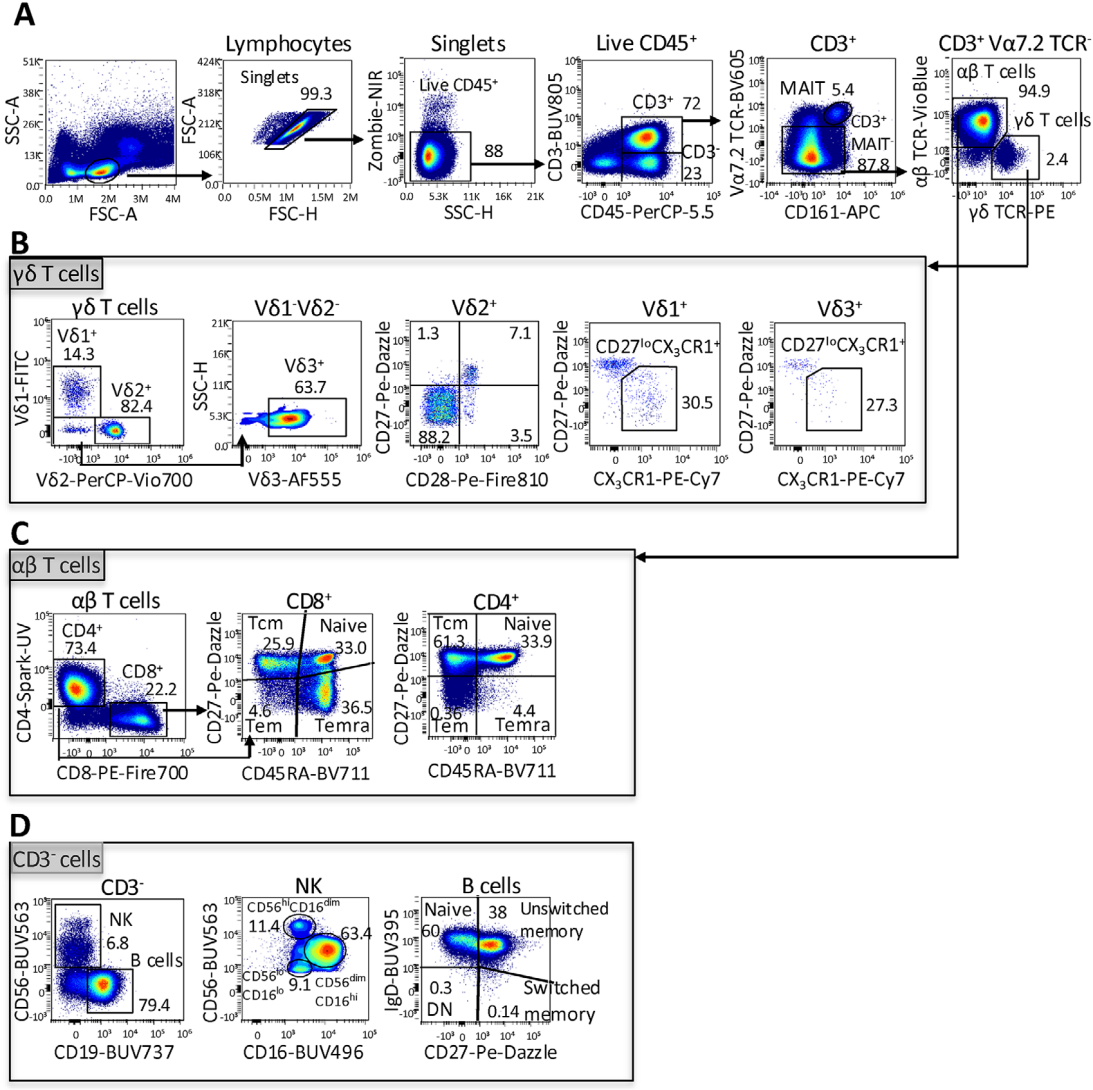
Gating strategy for γδ T cells and major lymphocyte subsets in PBMCs. (A) Lymphocytes were identified by FSC/SSC, doublets and dead cells excluded, and viable CD45^+^ cells gated. CD3^+^ cells were separated into MAIT (CD161^+^Vα7.2^+^), γδ T cells and αβ T cells. (B) γδ T cells were resolved into Vδ1, Vδ2 and Vδ3 subsets; Vδ2 cells were classified by CD27/CD28, and Vδ1/Vδ3 by CD27/CX_3_CR1. (C) CD4^+^ and CD8^+^ αβ T cells were subdivided into naïve, TCM, TEM and TEMRA using CD45RA/CD27. (D) CD3^−^ cells yielded NK cells (CD56^+^) and B cells (CD19^+^); NK subsets were defined by CD56/CD16, and B cells as naïve, unswitched memory, switched memory or DN by IgD/CD27. Data are representative of PBMCs from three independent healthy donors (*n* = 3).

To assess the panel's performance across lymphoid tissues, we evaluated its ability to resolve major γδ T cell subsets using representative gating and UMAP‐based dimensionality reduction. The panel successfully identified all principal γδ T cell subsets, Vδ1^+^, Vδ2^+^ and Vδ3^+^, in peripheral blood, spleen and bone marrow (Figure 2A). UMAP projections further revealed clear subset segregation and tissue‐specific clustering (Figure 2B). As expected, Vδ2^+^ T cells were predominantly enriched in blood, whereas Vδ1^+^ and Vδ3^+^ subsets displayed broader phenotypic diversity in spleen and bone marrow, consistent with tissue‐adapted profiles. These findings demonstrate the panel's utility for resolving rare and diverse γδ T cell populations across immune compartments.

**FIGURE 2:**
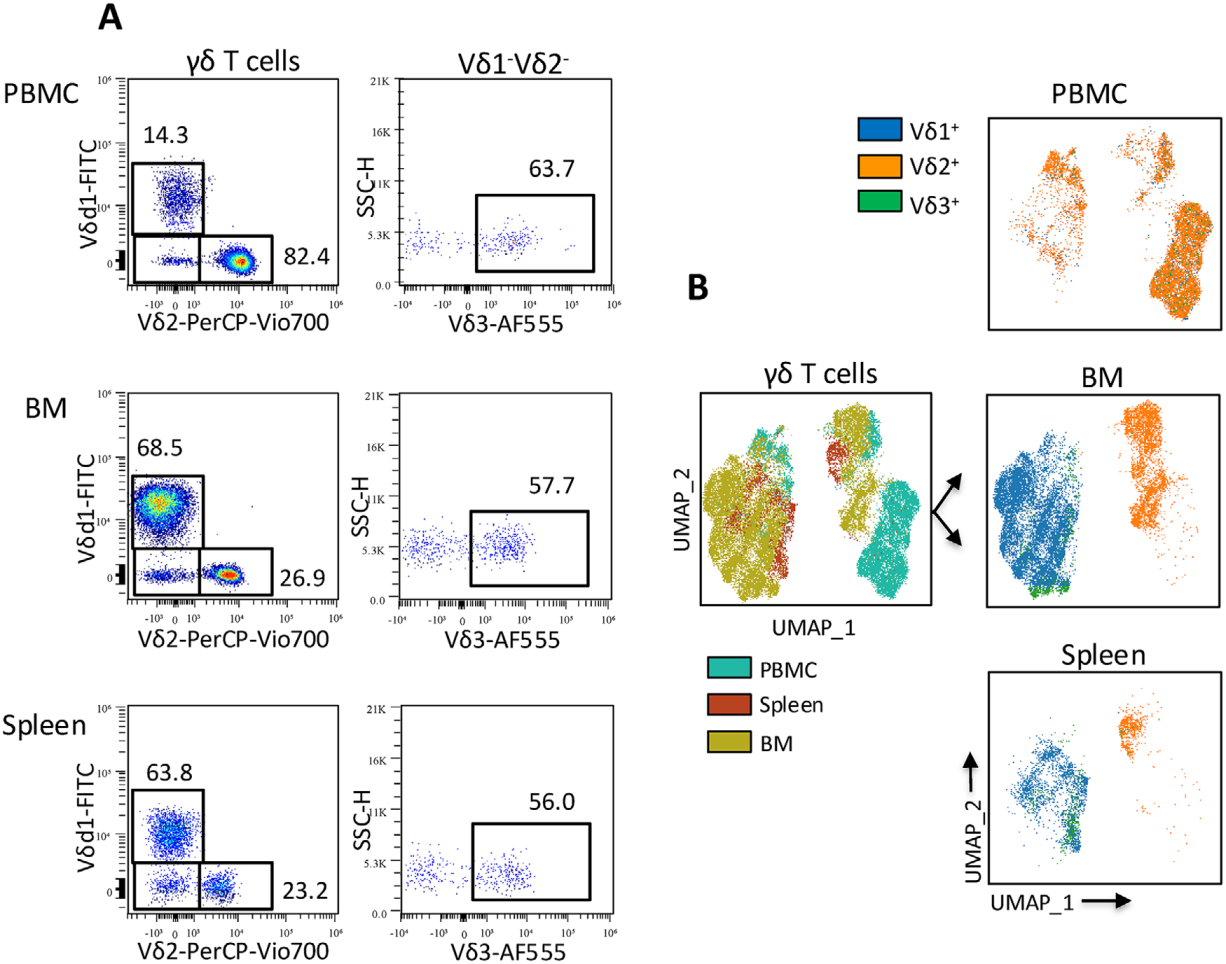
γδ T cell subset distribution and phenotypic diversity across tissues. (A) Representative flow cytometry plots show gating and relative proportions of Vδ1^+^, Vδ2^+^ and Vδ3^+^ γδ T cells in PBMCs, bone marrow and spleen, defined by specific TCR staining. (B) UMAP projection of concatenated γδ T cell events across tissues. Data shown are representative of PBMCs from three donors, spleen from two donors and bone marrow from two donors.

Consistent with the subset definitions shown in Figures 1 and 2, phenotypic profiling revealed clear differences between circulating Vδ2^+^ γδ T cells and tissue‐enriched Vδ1^+^ and Vδ3^+^ populations. Vδ2^+^ cells exhibited higher expression of CD28 and CD127, indicative of a naïve‐like phenotype, whereas Vδ1^+^ and Vδ3^+^ subsets showed reduced expression of these markers and increased expression of cytotoxic and inhibitory markers, including CD16, CD57 and PD‐1, particularly in spleen and bone marrow (Figure S1).

We next used this panel to characterise the rare and poorly characterised Vδ3^+^ γδ T cell subset. Vδ3^+^ T cells were detected in peripheral blood, spleen and bone marrow, enabling analysis of TCR usage and differentiation. In contrast to Vδ2^+^ cells, which consistently paired with Vγ9 across tissues, Vδ3^+^ cells showed Vγ9 pairing only in circulation, with no detectable pairing in spleen or bone marrow (Figure S2A). This pattern differed from that of Vδ1^+^ cells, which paired with Vγ9 in both the blood and the spleen. Phenotypically, circulating Vδ3^+^ cells displayed a naïve‐like profile, whereas tissue‐derived Vδ3^+^ cells adopted an effector‐like, cytotoxic phenotype resembling Vδ1^+^ rather than Vδ2^+^ cells (Figure S2B).

Together, these results show that Vδ2^+^ T cells predominated in circulation, whereas Vδ1^+^ and Vδ3^+^ populations were enriched in spleen and bone marrow, consistent with established tissue compartmentalisation [10]. UMAP‐based dimensionality reduction confirmed robust clustering of major γδ T cell subsets (Vδ1^+^, Vδ2^+^, and Vδ3^+^) with consistent architecture across biological replicates. Phenotypic profiling revealed subset‐specific differences in differentiation, cytotoxicity and tissue adaptation. Notably, systematic analysis of the poorly characterised Vδ3^+^ subset revealed restricted Vγ9 pairing confined to circulation and a shift towards a cytotoxic, tissue‐adapted phenotype in the spleen and bone marrow, aligning more closely with Vδ1^+^ than Vδ2^+^ cells. These findings highlight the panel's ability to resolve rare, tissue‐adapted γδ T cell populations.

While the panel was designed to resolve major lymphocyte populations, a limitation is the lack of definitive markers for invariant Natural Killer T (iNKT) cells, such as CD1d‐αGalCer tetramers. NKT‐like CD3^+^CD56^+^ cells can be identified but not fully resolved. Future expansions could incorporate iNKT‐specific reagents to broaden the scope of innate‐like T cell profiling. Additionally, while this study focused on lymphoid tissues, the panel is adaptable to non‐lymphoid compartments such as gut, lung or skin, including residency/homing markers (e.g., CLA, α4β7, CCR10). This flexibility supports applications in infection, cancer, vaccination, and tissue‐specific immunity.

In summary, this high‐dimensional panel enables robust and high‐resolution profiling of γδ T cells and major lymphocytes across blood and tissue. Thus, this panel is particularly valuable for applications requiring reliable resolution of rare γδ T cell subsets and for advancing translational studies of γδ T cell biology and tissue‐resident immunity.

## Author Contributions

Conceptualisation: M.S.D. and M.H. Data curation, formal analysis, investigation, methodology, project administration, and visualisation: MH; methodology; investigation or resources: R.S., D.M.V., D.A., A.B., E.M., M.F., P.C. Supervision, funding acquisition, methodology, project administration: M.S.D. Manuscript writing: M.H. wrote the manuscript, and all authors reviewed and approved the manuscript.

## Funding

This work was supported by a Royal Society Wolfson Fellowship (RSWF∖R2∖222002) and the U.S. Department of Defense Discovery Award (PR210753) to MSD. RS and AB were funded by a scholarship from the Medical Research Council (MRC) MRC‐funded Doctoral Training Partnership (DTP) in Interdisciplinary Biomedical Research (MR/R015910/1). E.M. was funded by an Australian Government Postgraduate Scholarship. D.M.V. was funded by a Monash Biomedicine Discovery Institute (BDI) Postgraduate Scholarship.

## Conflicts of Interest

The authors declare no conflicts of interest.

## Supporting information




**Supporting File**: eji70155‐sup‐0001‐SuppMat.pdf.

## Data Availability

The data supporting the findings of this study are available on request from the corresponding author.
