# Supplementary material for: 38‐Marker Full‐Spectrum Flow Cytometry Panel for the Comprehensive Profiling of γδ T Cells in Human Blood and Lymphoid Tissues
Source: Eur J Immunol. 2026 Mar 1;56(3):e70155. doi: 10.1002/eji.70155 (PMC12949950; doi:10.1002/eji.70155)
Supplement: Supplementary file 1 — Supporting File: eji70155‐sup‐0001‐SuppMat.pdf. [file EJI-56-e70155-s001.pdf]

38-marker full-spectrum flow cytometry panel for the comprehensive profiling of γδ T cells in human blood and lymphoid tissues

Authors: Mohamed Hamed[1], Daniela Moreno-Vicencio[1], Daniel Arsovski[1], Mustafa Farhat[1], Rosie Sanders[1], Ellen Mann[1], Annabelle Bennett[1], Priyanka Chevour[1] and Martin S. Davey[1,2*]

Affiliations:
[1]Division of Biomedical Sciences, Warwick Medical School, University of Warwick, Coventry, UK
[2]Infection and Immunity Program and Department of Biochemistry and Molecular Biology, Biomedicine Discovery Institute, Monash University, Clayton, Victoria 3800, Australia.

Supplementary Materials and Methods

Sample Collection and Processing

Human peripheral blood mononuclear cells (PBMC) (*n*=3) and bone marrow (*n*=2) from healthy adult donors were obtained from STEMCELL Technologies (Vancouver, Canada) and approved by the University of Warwick Biomedical and Scientific Research Ethics Committee (BSREC) #90/23-24. Spleen samples (*n*=2) were obtained from the University Hospital Coventry and Warwickshire NHS Trust Arden Tissue Biobank (Coventry, UK) #ATB22-10. All human samples were collected according to ethical standards set by the Declaration of Helsinki.

Panel Design and Fluorochrome Selection

The 38-marker spectral panel for profiling of γδ T cells in tissues was developed based on previous work [5, 10], with the goal of maximising resolution of γδ T cells and other lymphocyte subsets across multiple tissue types. Panel design followed best practices outlined in the third edition of the Flow Cytometry Guidelines [1]. Fluorochromes were selected based on their spectral separation, brightness, and minimal spillover, using the Sony ID7000 spectral reference data with 5 lasers as a guide. Low-expression markers were paired with high-brightness dyes to enhance signal detection, while co-expressed antigens were assigned spectrally distinct fluorochromes to minimise spreading error. Priority was given to well-validated antibody clones. The final configuration included markers for lineage identification,

T cell receptor usage, differentiation, activation, and tissue residency. The complete antibody panel, including fluorochrome assignments, is provided in Table S1.

Development of the 38-marker spectral panel involved iterative refinement of marker-fluorochrome assignments and individual antibody titrations, followed by comprehensive testing across human PBMCs, spleen, and bone marrow to ensure optimal resolution and reproducibility. A theoretical assessment of marker-fluorochrome assignments using the Cytek panel viewer for similarity and complexity indices yielded a similarity score of 33.8 for the 38-color panel (Figure S3), indicating efficient marker distribution and moderate predicted spectral interference relative to other high-dimensional panel designs [5]. This was balanced by assigning fluorochromes with high similarity indices to markers with non-overlapping expression, such as CD3 and CD19.

Antibody Titration Strategy

Antibody titrations were performed using thawed PBMCs under staining conditions identical to those used in the full 38-marker panel. Primary antibodies targeting major lineage-defining markers (e.g., CD3, CD19, CD56) were titrated individually, and the optimal staining concentration was selected based on the best separation between the negative and the positive populations (Figure S4A). For low-expression or continuous-expression antigens (e.g., CCR6), titrations were carried out in the presence of relevant lineage markers to enable gating within defined populations and assess signal resolution in the appropriate biological context (Figure S4B). Two antibodies required further optimisation, CD161 and CCR7. CCR7 was stained separately prior to adding the rest of the antibody cocktail (Figure S4C). CD161 signal was improved after switching from APC-Fire-750 to APC (Figure S4D). Optimal titres were determined by achieving the maximum separation between positive and negative populations while minimising background fluorescence using the stain index calculated as

$$\text{Stain Index (SI)} = (\text{MFI}\_{\text{positive}} - \text{MFI}\_{\text{negative}}) / (2 \times \text{SD}\_{\text{negative}})$$

The final selection of reagents and titres shows reliable identification of populations of interest in blood and tissues. Clone selection prioritised reagents with prior validation in human samples, and marker redundancy was minimized to streamline gating.
Titrations were performed using serial twofold dilutions across multiple points.

Spectral Staining Procedure and Sample Acquisition

Cryopreserved PBMCs, bone marrow and spleen were rapidly thawed at 37°C and diluted in 10 mL in complete RPMI (RPMI-1640 supplemented with 10% FCS, 0.3 g/L L-glutamine, 5mM Sodium Pyruvate, 5mM Non-essential amino acids, 5mM HEPES and 1% penicillin-streptomycin) (Thermo Fisher). Cells were centrifuged at 400 × g for 5 minutes at 4°C, followed by an additional wash in medium and filtration through a 70-μm cell strainer. Cell recovery was assessed using Trypan Blue, and between 3-5 million viable cells per sample were used for staining. Cells were stained with Zombie NIR fixable viability dye (1:400; BioLegend) in PBS for 10 minutes in the dark at room temperature. After washing with fluorescence-activated cell sorting (FACS) buffer (sterile PBS, 2% FBS, and 0.04% sodium azide), cells were then stained with a 50 μL antibody cocktail prepared in Brilliant Stain Buffer (BD Biosciences), supplemented with 1% Fc receptor blocking reagent (Miltenyi Biotec), and incubated for 20 minutes at 4°C. Following surface staining, cells were washed, resuspended in FACS buffer, and kept on ice in the dark until acquisition on the flow cytometer. All samples were acquired on a 5-laser Sony ID7000 spectral flow cytometer (Warwick Medical School) using factory-optimised instrument settings.

Spectral Flow Cytometry and Data Analysis

Quality control and spectral reference checks were performed using Spectral Calibration and QC Beads, following the manufacturer's instructions. Forward and side scatter settings were adjusted to optimise lymphocyte identification, and all samples were acquired at a low flow rate. Spectral unmixing was conducted using single-stained UltraComp eBeads (Invitrogen) and matched single-stained cells as internal controls. Data analysis was carried out using FlowJo v10.10 (BD Biosciences) and OMIQ (Dotmatics), with manual gating applied to define lymphocyte subsets. Dimensionality reduction was performed using UMAP in OMIQ, based on approximately 10,000 $CD3^{+}$ $\gamma\delta TCR^{+}$ events per sample per tissue. UMAP settings included Euclidean distance, 15 nearest neighbors, a minimum distance of 0.4, and projection into 2 dimensions. Input parameters included phenotypic and lineage markers: Vδ1, Vδ2, Vδ3, γδTCR, CD3, CD11a, CD16, CD25, CD56, CD57, HLA-DR, CCR7, CD45RA, CD45RO, CD69, CXCR3, CD103, DNAM-1, CD127, Vγ9, CD161, CCR6, CD38, and CCR5. Markers used solely for gating or viability (e.g., FSC, SSC, CD45, and live/dead dyes) were excluded from dimensionality reduction. Prior to UMAP, data were normalised using the Sony ID7000 built-in scaling and unmixing tools to reduce technical variability across samples. Batch effects arising from acquisition day or tissue source were addressed using

Harmony batch correction in OMIQ, enabling effective alignment of shared cell populations while preserving biological variation

Pitfalls and Tricks

Anti-Vδ3 detection: A validated in-house AF555-conjugated anti-Vδ3 monoclonal antibody was essential for reliable identification of Vδ3^{+} cells. This reagent is now widely available from Miltenyi Biotec (Clone: REA1426 | P11.5B) conjugated to different fluorophores, but care should be taken to integrate this antibody into the panel, as we did not test this here.

CCR7 signal quality: Staining CCR7 separately before adding the cocktail improved signal resolution and reproducibility.

Marker redundancy: Avoid excessive inclusion of overlapping markers. For example, CD45RA/CD45RO alone was sufficient to resolve memory subsets when combined with CD27.

CD161 clone choice: Switching from APC-Fire750 to APC dramatically improved staining quality and resolution.

Signal spreading from highly expressed markers (e.g., CD3, CD45): These should be assigned to less-sensitive channels to reduce spreading error for low-expression markers.

Laser	Fluorophore	Antigen	Clone	Supplier	Catalogue No.	Dilution
**UV - 355nm**	Spark UV387	CD4	SK3	Biolegend	344685	1:200
	BUV395	IgD	Clone IA6-2	BD	563813	1:100
	BUV496	CD16	3G8	BD	612945	1:100
	BUV563	CD56	NCAM16.2	BD	612928	1:100
	BUV615	CD25	2A3	BD	612997	1:100
	BUV661	CD11a	G-25.2	BD	750270	1:400
	BUV737	CD19	SJ25C1	BD	612757	1:100
	BUV805	CD3	UCHT1	BD	612896	1:30
**V - 405nm**	BV421	PD-1	EH12.1	Biolegend	329920	1:50
	BV480	CD94	HP-3D9	BD	746737	1:200
	VioBlue	αβTCR	REA652	Miltenyi	130-119-618	1:100
	BV510	CD57	QA17A04	Biolegend	393313	1:100
	BV570	HLA-DR	L243	Biolegend	307637	1:100
	BV605	Va7.2	3C10	Biolegend	351719	1:30
	BV650	CCR7	G043H7	Biolegend	353233	1:30
	BV711	CD45RA	HI100	Biolegend	304137	1:200
	BV750	CD69	FN50	Biolegend	310953	1:100
	BV786	CXCR3	G025H7	Biolegend	353737	1:50
**B - 488nm**	FITC	Vδ1	REA173	Miltenyi	130-118-362	1:200
	RB545	CXCR5	RF8B2	BD	756520	1:200
	RB780	DNAM-1	DX11	BD	755549	1:100
	BB700	CD103	Ber-ACT8	BD	745919	1:100
	PeCP Cy5.5	CD45	HI30	Biolegend	304028	1:200
	PerCP Vio700	Vδ2	REA771	Miltenyi	130-111-014	1:50
	AF555	Vδ3	P11.5B	Beckman	IM1563	1:200
**YG - 561nm**	PE	γδTCR	REA591	Miltenyi	130-113-512	1:200
	Spark YG 581	IL-7Rα	A019D5	Biolegend	351367	1:100
	PE-Dazzle594	CD27	M-T271	Biolegend	356422	1:200
	PE-Cy5	Vγ9	IMMU 360	Beckman	304074	1:400
	PE-Cy7	CX_3CR1	2A9-1	Biolegend	341612	1:100
	Pe-Fire 700	CD8a	SK1	Biolegend	344766	1:100
	PE-Fire 810	CD28	CD28.2	Biolegend	302971	1:50
**Red - 637nm**	APC	CD161	W18070C	Biolegend	307511	1:100
	AF 647	CCR6	11A9	BD	560466	1:50
	R718	CD45RO	UCHL1	BD	567536	1:100
	APC-Fire750	CCR5	J418F1	Biolegend	359133	1:50
	Zombie NIR	Live/Dead	-	Biolegend	423117	1:400
	APC-Fire 810	CD38	HIT2	Biolegend	303550	1:200

Supplementary Table S1. Antibodies, fluorochromes, clones, and titration details for the 38-marker panel.

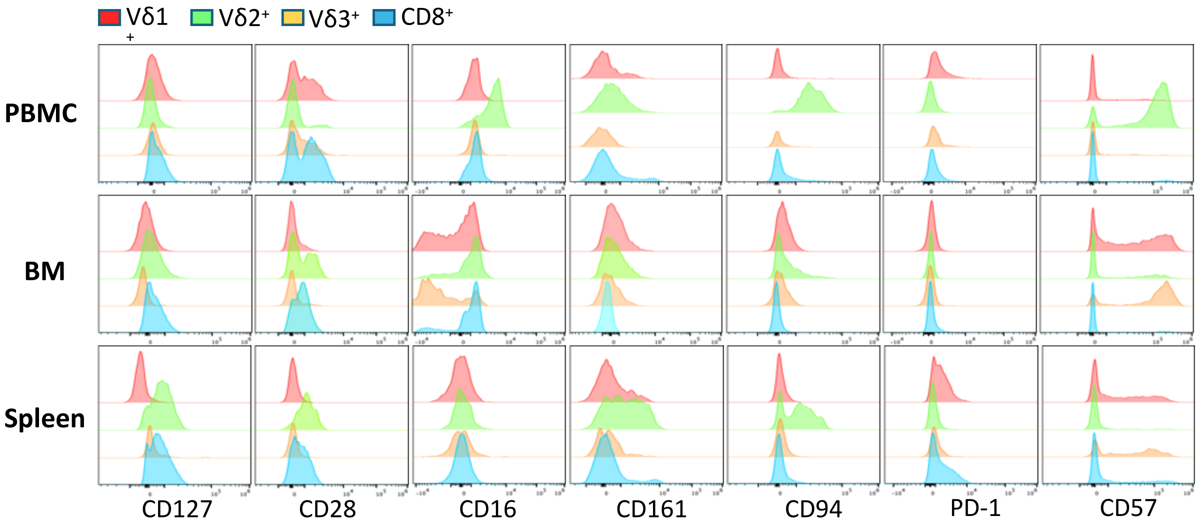

Supplementary Figure S1. Phenotypic marker distribution across γδ T cell subsets. Histograms illustrate the differential expression of phenotypic markers across γδ T cell subsets in PBMCs, bone marrow and spleen. Data shown are representative of PBMCs (n = 3), spleen (n = 2), and bone marrow (n = 2) samples.

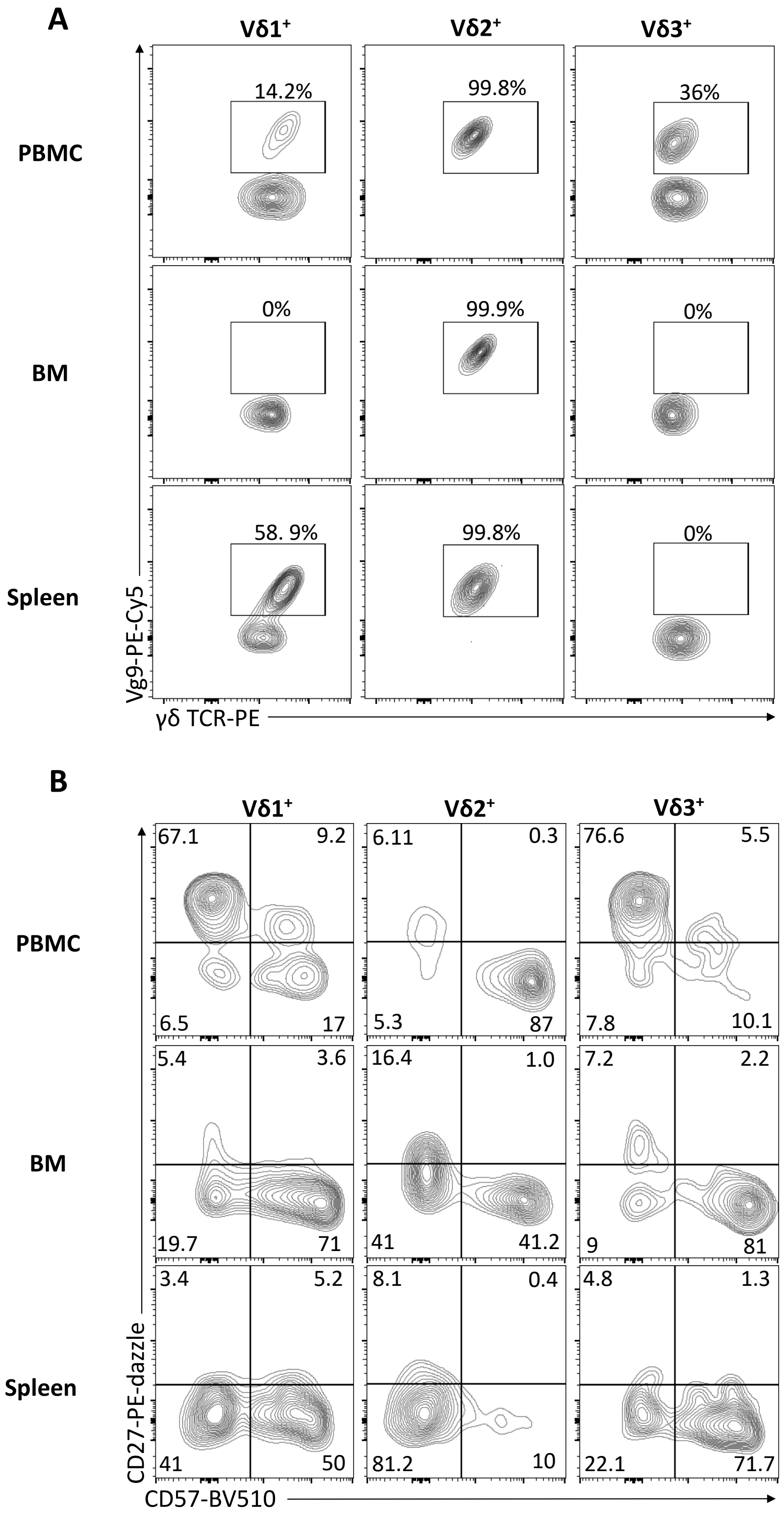

Supplementary Figure S2. Vδ3⁺ γδ T cells exhibit tissue-specific differentiation profiles distinct from Vδ1⁺ and Vδ2⁺ γδ T cell subsets. (**A**) Co-expression analysis of γδ TCR and Vγ9 within gated Vδ1⁺, Vδ2⁺, and Vδ3⁺ γδ T cells from PBMCs, bone marrow, and spleen. Percentages indicate the frequency of Vγ9 pairing within each subset. (**B**) Contour plots showing CD27 and CD57 expression on gated CD3⁺ Vδ1⁺, Vδ2⁺, and Vδ3⁺ γδ T cells from PBMCs, bone marrow, and spleen. Data shown are representative of PBMCs (n = 3), spleen (n = 2), and bone marrow (n = 2) samples.

A

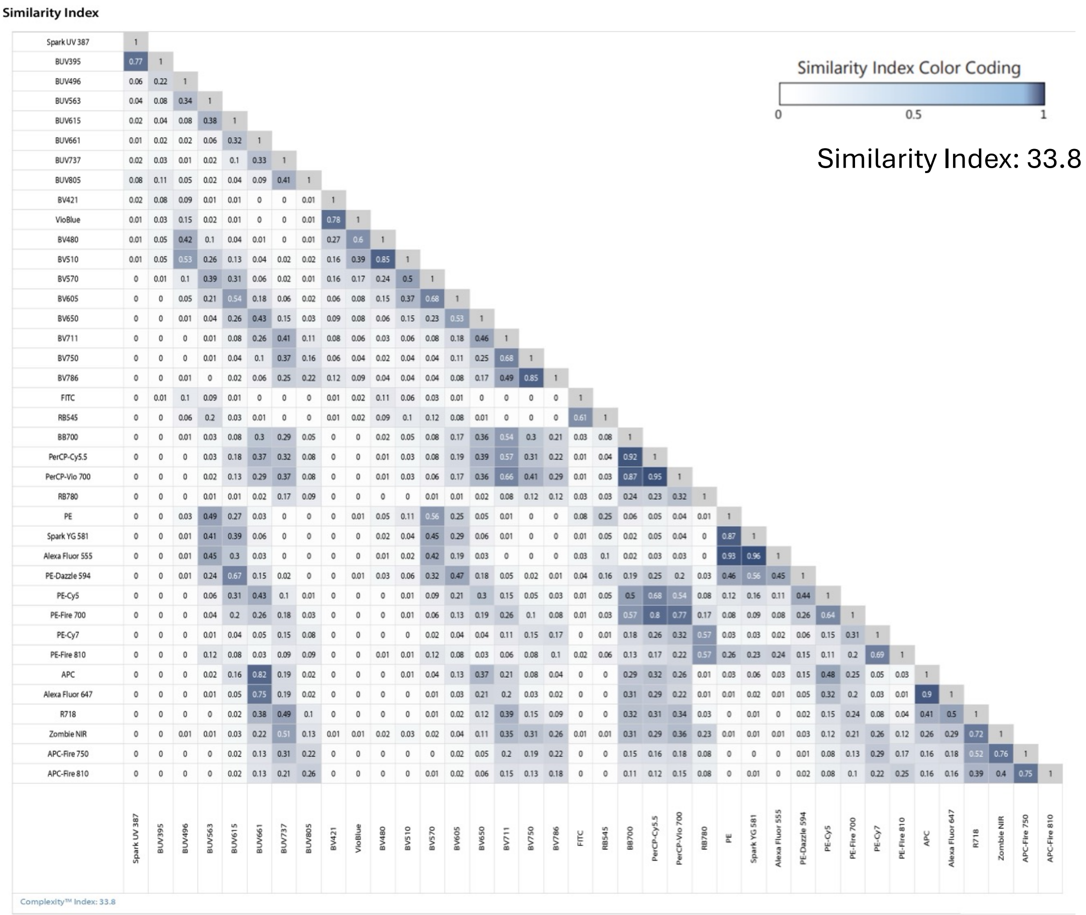

	Spark UV 387	BUV395	BUV496	BUV563	BUV615	BUV661	BUV737	BUV805	BV421	VioBlue	BV480	BV510	BV570	BV605	BV650	BV711	BV750	BV786	FITC	RB545	BB700	PerCP-Cy5.5	PerCP-Vio 700	RB780	PE	Spark YG 581	Alexa Fluor 555	PE-Dazzle 594	PE-Cy5	PE-Fire 700	PE-Cy7	PE-Fire 810	APC	Alexa Fluor 647	R718	Zombie NIR	APC-Fire 750	APC-Fire 810
Spark UV 387	1																																					
BUV395	0.77	1																																				
BUV496	0.06	0.22	1																																			
BUV563	0.04	0.08	0.34	1																																		
BUV615	0.02	0.04	0.08	0.38	1																																	
BUV661	0.01	0.02	0.02	0.06	0.32	1																																
BUV737	0.02	0.03	0.01	0.02	0.1	0.33	1																															
BUV805	0.08	0.11	0.05	0.02	0.04	0.09	0.41	1																														
BV421	0.02	0.08	0.09	0.01	0.01	0	0	0.01	1																													
VioBlue	0.01	0.03	0.15	0.02	0.01	0	0	0.01	0.78	1																												
BV480	0.01	0.05	0.42	0.1	0.04	0.01	0	0.01	0.27	0.6	1																											
BV510	0.01	0.05	0.53	0.26	0.13	0.04	0.02	0.02	0.16	0.39	0.85	1																										
BV570	0	0.01	0.1	0.39	0.31	0.06	0.02	0.01	0.16	0.17	0.24	0.5	1																									
BV605	0	0	0.05	0.21	0.54	0.18	0.06	0.02	0.06	0.08	0.15	0.37	0.68	1																								
BV650	0	0	0.01	0.04	0.26	0.43	0.15	0.03	0.09	0.08	0.06	0.15	0.23	0.53	1																							
BV711	0	0	0	0.01	0.08	0.26	0.41	0.11	0.08	0.06	0.03	0.06	0.08	0.18	0.46	1																						
BV750	0	0	0	0.01	0.04	0.1	0.37	0.16	0.06	0.04	0.02	0.04	0.04	0.11	0.25	0.68	1																					
BV786	0	0	0.01	0	0.02	0.06	0.25	0.22	0.12	0.09	0.04	0.04	0.04	0.08	0.17	0.49	0.85	1																				
FITC	0	0.01	0.1	0.09	0.01	0	0	0	0.01	0.02	0.11	0.06	0.03	0.01	0	0	0	0	1																			
RB545	0	0	0.06	0.2	0.03	0.01	0	0	0.01	0.02	0.09	0.1	0.12	0.08	0.01	0	0	0	0.61	1																		
BB700	0	0	0.01	0.03	0.08	0.3	0.29	0.05	0	0	0.02	0.05	0.08	0.17	0.36	0.54	0.3	0.21	0.03	0.08	1																	
PerCP-Cy5.5	0	0	0	0.03	0.18	0.37	0.32	0.08	0	0	0.01	0.03	0.08	0.19	0.39	0.57	0.31	0.22	0.01	0.04	0.92	1																
PerCP-Vio 700	0	0	0	0.02	0.13	0.29	0.37	0.08	0	0	0.01	0.03	0.06	0.17	0.36	0.66	0.41	0.29	0.01	0.03	0.87	0.95	1															
RB780	0	0	0	0.01	0.01	0.02	0.17	0.09	0	0	0	0	0.01	0.01	0.02	0.08	0.12	0.12	0.03	0.03	0.24	0.23	0.32	1														
PE	0	0	0.03	0.49	0.27	0.03	0	0	0	0.01	0.05	0.11	0.56	0.25	0.05	0.01	0	0	0.08	0.25	0.06	0.05	0.04	0.01	1													
Spark YG 581	0	0	0.01	0.41	0.39	0.06	0	0	0	0	0.02	0.04	0.45	0.29	0.06	0.01	0	0	0.01	0.05	0.02	0.05	0.04	0	0.87	1												
Alexa Fluor 555	0	0	0.01	0.45	0.3	0.03	0	0	0	0	0.01	0.02	0.42	0.19	0.03	0	0	0	0.03	0.1	0.02	0.03	0.03	0	0.93	0.96	1											
PE-Dazzle 594	0	0	0.01	0.24	0.67	0.15	0.02	0	0	0.01	0.03	0.06	0.32	0.47	0.18	0.05	0.02	0.01	0.04	0.16	0.19	0.25	0.2	0.03	0.46	0.56	0.45	1										
PE-Cy5	0	0	0	0.06	0.31	0.43	0.1	0.01	0	0	0	0.01	0.09	0.21	0.3	0.15	0.05	0.03	0.01	0.05	0.5	0.68	0.54	0.08	0.12	0.16	0.11	0.44	1									
PE-Fire 700	0	0	0	0.04	0.2	0.26	0.18	0.03	0	0	0	0.01	0.06	0.13	0.19	0.26	0.1	0.08	0.01	0.03	0.57	0.8	0.77	0.17	0.08	0.09	0.08	0.26	0.64	1								
PE-Cy7	0	0	0	0.01	0.04	0.05	0.15	0.08	0	0	0	0	0.02	0.04	0.04	0.11	0.15	0.17	0	0.01	0.18	0.26	0.32	0.57	0.03	0.03	0.02	0.06	0.15	0.31	1							
PE-Fire 810	0	0	0	0.12	0.08	0.03	0.09	0.09	0	0	0.01	0.01	0.12	0.08	0.03	0.06	0.08	0.1	0.02	0.06	0.13	0.17	0.22	0.57	0.26	0.23	0.24	0.15	0.11	0.2	0.69	1						
APC	0	0	0	0.02	0.16	0.82	0.19	0.02	0	0	0	0.01	0.04	0.13	0.37	0.21	0.08	0.04	0	0	0.29	0.32	0.26	0.01	0.03	0.06	0.03	0.15	0.48	0.25	0.05	0.03	1					
Alexa Fluor 647	0	0	0	0.01	0.05	0.75	0.19	0.02	0	0	0	0	0.01	0.03	0.21	0.2	0.03	0.02	0	0	0.31	0.29	0.22	0.01	0.01	0.02	0.01	0.05	0.32	0.2	0.03	0.01	0.9	1				
R718	0	0	0	0	0.02	0.38	0.49	0.1	0	0	0	0	0.01	0.02	0.12	0.39	0.15	0.09	0	0	0.32	0.31	0.34	0.03	0	0.01	0	0.02	0.15	0.24	0.08	0.04	0.41	0.5	1			
Zombie NIR	0	0	0.01	0.01	0.03	0.22	0.51	0.13	0.01	0.01	0.02	0.03	0.02	0.04	0.11	0.35	0.31	0.26	0.01	0.01	0.31	0.29	0.36	0.23	0.01	0.01	0.01	0.03	0.12	0.21	0.26	0.12	0.26	0.29	0.72	1		
APC-Fire 750	0	0	0	0	0.02	0.13	0.31	0.22	0	0	0	0	0	0.02	0.05	0.2	0.19	0.22	0	0	0.15	0.16	0.18	0.08	0	0	0	0.01	0.08	0.13	0.29	0.17	0.16	0.18	0.52	0.76	1	
APC-Fire 810	0	0	0	0	0.02	0.13	0.21	0.26	0	0	0	0	0.01	0.02	0.06	0.15	0.13	0.18	0	0	0.11	0.12	0.15	0.08	0	0.01	0	0.02	0.08	0.1	0.22	0.25	0.16	0.16	0.39	0.4	0.75	1

B

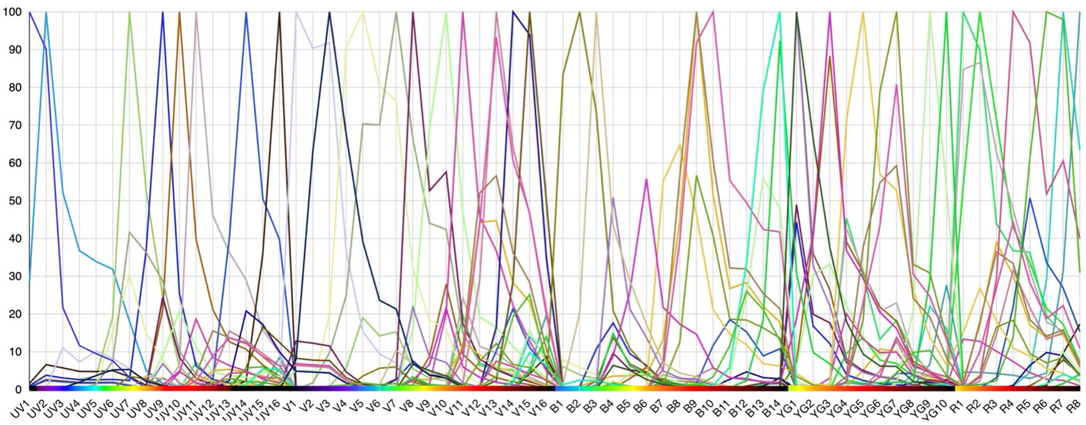

Supplementary Figure S3. Fluorochrome Similarity Matrix and Spectral Overlay of the 38-Color Panel. (**A**) Similarity index matrix displaying numerical values for each pairwise comparison of fluorochromes selected for the 38-color panel. (**B**) Spectral signatures of the 38 fluorochromes used in the panel were overlaid using the Cytek Cloud.

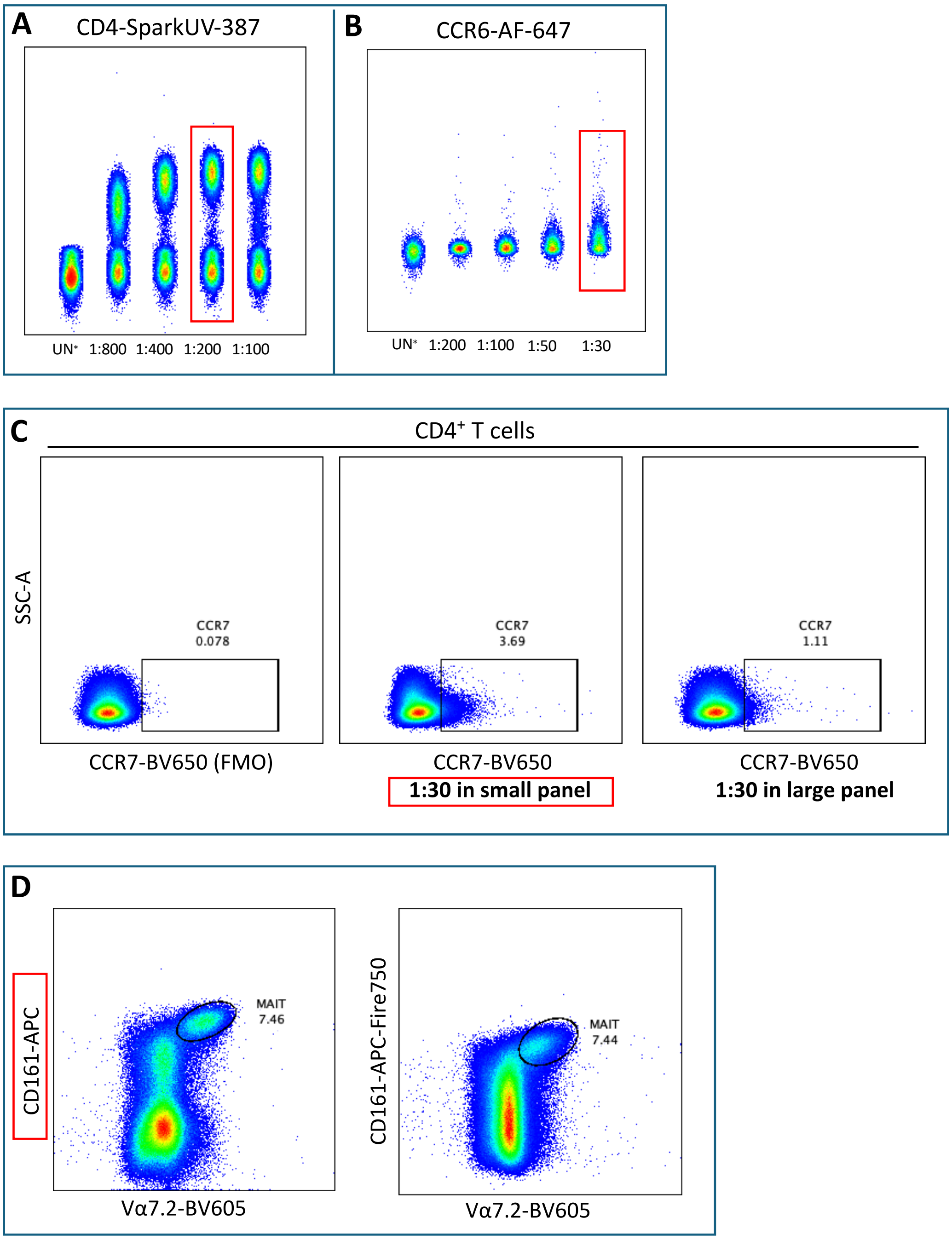

Supplementary Figure S4. Antibody titration and optimisation steps for panel development.
All antibodies were titrated using a 2-fold serial dilution in 50 µL per test. **(A)** CD4-SparkUV 387 titration: a representative example of primary markers titrated individually. The dilution providing the best separation between negative and positive populations was selected. **(B)** CCR6-AF647 titration: Low-expression antigens such as CCR6 were titrated in the presence of other antibodies to enable accurate gating of specific cell subsets. The concentration with optimal resolution was chosen. **(C)** CCR7-BV650 staining: Pre-staining CCR7 before adding the rest of the antibody panel improved signal quality and resolution. **(D)** Switching CD161 from APC-Fire750 to APC enhanced the resolution of MAIT cell identification.
